# Global patterns of submicroscopic *Plasmodium falciparum* malaria infection: insights from a systematic review and meta-analysis of population surveys

**DOI:** 10.1016/S2666-5247(21)00055-0

**Published:** 2021-08

**Authors:** Charles Whittaker, Hannah Slater, Rebecca Nash, Teun Bousema, Chris Drakeley, Azra C Ghani, Lucy C Okell

**Affiliations:** a MRC Centre for Global Infectious Disease Analysis, Department of Infectious Disease Epidemiology, Imperial College London, London, UK; b PATH, Seattle, WA, USA; c Department of Medical Microbiology, Radboud University Medical Center, Nijmegen, Netherlands; d Department of Infection Biology, Faculty of Infectious and Tropical Diseases, London School of Hygiene & Tropical Medicine, London, UK

## Abstract

**Background:**

Adoption of molecular techniques to detect *Plasmodium falciparum* infection has revealed many previously undetected (by microscopy) yet transmissible low-density infections. The proportion of these infections is typically highest in low transmission settings, but drivers of submicroscopic infection remain unclear. Here, we updated a previous systematic review of asexual *P falciparum* prevalence by microscopy PCR in the same population. We aimed to explore potential drivers of submicroscopic infection and to identify the locations where submicroscopic infections are most common.

**Methods:**

In this systematic review and meta-analysis we searched PubMed and Web of Science from Jan 1, 2010, until Oct 11, 2020, for cross-sectional studies reporting data on asexual *P falciparum* prevalence by both microscopy and PCR. Surveys of pregnant women, surveys in which participants had been chosen based on symptoms or treatment, or surveys that did not involve a population from a defined location were excluded. Both the number of individuals tested and the number of individuals who tested positive by microscopy or PCR, or both, for *P falciparum* infection were extracted. Bayesian regression modelling was used to explore determinants of the size of the submicroscopic reservoir including geographical location, seasonality, age, methodology, and current or historical patterns of transmission.

**Findings:**

Of 4893 identified studies, we retained 121 after screening and removal of duplicates. 45 studies from a previous systematic review were included giving 166 studies containing 551 cross-sectional survey microscopy and PCR prevalence pairs. Our results show that submicroscopic infections predominate in low-transmission settings across all regions, but also reveal marked geographical variation, with the proportion of infections that are submicroscopic being highest in South American surveys and lowest in west African surveys. Although current transmission levels partly explain these results, we find that historical transmission intensity also represents a crucial determinant of the size of the submicroscopic reservoir, as does the demographic structure of the infected population (with submicroscopic infection more likely to occur in adults than in children) and the PCR or microscopy methodology used. We also observed a small yet significant influence of seasonality, with fewer submicroscopic infections observed in the wet season than the dry season. Integrating these results with estimates of infectivity in relation to parasite density suggests the contribution of submicroscopic infections to transmission across different settings is likely to be highly variable.

**Interpretation:**

Significant variation in the prevalence of submicroscopic infection exists even across settings characterised by similar current levels of transmission. These differences in submicroscopic epidemiology potentially warrant different approaches to targeting this infected subgroup across different settings to eliminate malaria.

**Funding:**

Bill & Melinda Gates Foundation, The Royal Society, and the UK Medical Research Council.

## Introduction

The ability to accurately detect malaria infection during population surveys is a cornerstone of effective surveillance and control of the *Plasmodium* parasite. Routinely, malaria detection is undertaken using microscopy of blood films or rapid diagnostic tests, although in recent years there has been an increase in the use of more sensitive molecular methods in research contexts. These techniques (typically PCR based)^1^ have revealed the widespread presence of infections with parasite densities lower than the threshold of detection by routine methods such as microscopy.^2^, ^3^, ^4^ Such submicroscopic infections are present across a range of different settings and populations.^5^, ^6^ Although rarely causative of severe symptoms, these infections have been associated with some adverse outcomes during pregnancy^7^ and in children younger than 10 years.^8^

These infections are also relevant to public health because of their potential to be transmittable, despite being undetectable by conventional diagnostics. Although typically characterised by lower parasite densities and infectivity than microscopically detectable infections,^9^ individuals with submicroscopic infections frequently harbour gametocytes (the transmissible form of the parasite) and can contribute to onwards transmission of malaria. Individuals with submicroscopic infections have been shown to contribute to transmission across areas of high^10^ and low^11^ transmission intensity, as well as seasonal^12^ and perennial^13^ settings, underscoring the potential relevance of this infection subgroup to malaria control efforts.

**Research in context**

**Evidence before this study**

Cross-sectional surveys of malaria prevalence in endemic populations have revealed the widespread presence of infections with parasite densities lower than the threshold of detection by microscopy. These submicroscopic infections can infect mosquitoes and contribute to onwards transmission; therefore, understanding when and where these infections are most common is vital to the control of malaria transmission. A previous systematic review of these surveys highlighted that the fraction of *Plasmodium falciparum* infections missed by microscopy can be substantial (approximately 45% of all infections detected by PCR on average), that submicroscopic infection is more frequent in adults than in children (a phenomenon attributed to increased levels of immunity in adults and the associated suppression of parasite densities by this immune response), and that this missed fraction (ie, the submicroscopic reservoir) is typically highest in areas with low transmission intensity.

**Added value of this study**

The results presented here support previous research linking submicroscopic infection to older individuals and to areas where current transmission levels are low. However, the increased availability of data used in our study allows a richer and more complex characterisation of variation in submicroscopic epidemiology between settings. Our findings highlight marked geographical variation in the size of the submicroscopic reservoir, which cannot be explained by current transmission levels and instead appear primarily to be driven by the historical patterns of transmission in a setting. We also identify both seasonality and variation in diagnostic quality as drivers of submicroscopic infection prevalence across the population. Integrating these results with estimates of infectivity in relation to parasite density suggests the contribution of these submicroscopic infections to transmission across different settings is likely to be highly variable.

**Implications of all the available evidence**

Our work highlights material differences in submicroscopic malaria epidemiology across settings and suggests the absence of a one-size-fits-all solution for malaria control efforts targeting this infected subgroup. These differences likely warrant different and setting-specific approaches to targeting the submicroscopic reservoir if malaria is to be most effectively controlled in the approach to elimination.

Despite this potential relevance to malaria transmission, our understanding of the factors influencing the size of the submicroscopic reservoir remains far from complete. Previous reviews have found that microscopy misses on average, half of all *Plasmodium falciparum* infections compared with PCR-based methods in cross-sectional surveys^14^ and that adults are more likely to harbour submicroscopic infections than children.^15^ However, these reviews also identified extensive unexplained variation in the size of the submicroscopically infected population across settings, suggesting the existence of other important factors that determine the size of the reservoir. For example, although the extent of submicroscopic infection is highly heterogeneous across different locations,^5^, ^16^ it remains unclear whether this represents systematic variation according to geographical location or is reflective of other underlying location-specific characteristics.

Resolving these gaps in our understanding of submicroscopic epidemiology has material consequences for the future of malaria control. Given that submicroscopic infections can contribute to malaria transmission, understanding when and where they are most prevalent is vital to the control of the disease. Despite mixed reports surrounding more recent progress,^17^ malaria transmission is still declining in many endemic countries and elimination remains a target for a number of these. Low-transmission settings such as these can have high proportions of submicroscopically infected individuals.^18^, ^19^ Understanding the prevalence, detectability, and infectiousness of low-density infections in these settings will be essential for planning for the elimination of malaria: is there benefit in detecting and treating such infections, or are resources better spent elsewhere? Improving our understanding of the drivers of submicroscopic infection in these areas is, therefore, crucial to better define when and where submicroscopic infection is likely to occur and how the size of the submicroscopic reservoir is likely to change as areas approach elimination.

Here we update previous reviews on submicroscopic malaria infection prevalence,^14^, ^18^ leveraging the increase in the usage of molecular methods over the past decade to explore novel determinants of submicroscopic infection prevalence. These determinants include geographical location and historical patterns of transmission, diagnostic methodology, seasonality, and the role of age at a finer resolution than previously possible. These results are then integrated with estimates of the infectivity of submicroscopic individuals to mosquitoes to estimate their contribution to malaria transmission across a range of different settings.

## Methods

### Search strategy and selection criteria

We did a systematic review and meta-analysis of available data on submicroscopic malaria prevalence. Cross-sectional malaria prevalence data in which both microscopy and PCR based methods had been used to determine infection were compiled, updating a previous review published in 2012.^18^ PubMed and Web of Science were searched (by CW) using the terms “PCR” OR “Polymerase Chain Reaction” AND “falciparum” from Jan 1, 2010, (ie, the end date of the previous systematic review^18^) to Oct 11, 2020. Only studies in English were included. Studies reporting asexual *P falciparum* prevalence by microscopy and PCR in the same population were included. Surveys of pregnant women, in which participants were chosen on the basis of symptoms or treatment, or which did not involve a population from a defined location were excluded. Submicroscopic infections were defined as where infection was detectable by PCR but not by microscopy. Specificity of microscopy compared to PCR is high (average 98·4%^14^), so we assumed that microscopy-positive individuals are also PCR-positive.

### Data extraction

From each study, we extracted information on the number of individuals tested by PCR and microscopy, as well as the number of tested individuals positive for *P falciparum* malaria by each method. We also extracted data on the exact diagnostic methodologies used (specifically, the PCR method used and number of microscopy slides scanned) and characteristics of the survey location and timing (the global region, country, specific location, and sampling season). Where available, information on the age range of survey participants were also extracted.

### ANOVA and Tukey's honest significant difference

Data were analysed using an ANOVA based approach to assess differences in the mean prevalence ratio (defined as the proportion of PCR positive infections also detectable by light microscopy) and the factors underlying these differences. Data were weighted according to the cross-sectional survey sample size and controlling for the PCR prevalence recorded in the survey. Tukey's honest significant difference (HSD) test was used to post-hoc examine pairs of factors for significant differences in the mean prevalence ratio. These analyses were done using R statistical software, version 4.0.2.

### Bayesian log-linear regression

In line with a previous review,^18^ data were also analysed using the regression-based methodology, described by Sharp and Thompson,^20^ to estimate microscopy prevalence and the prevalence ratio as a function of PCR prevalence:

$${\text{LM}}_{i}={\text{PCR}}_{i}+\delta_{i}$$

where LM_i_ is the log odds of microscopy prevalence in survey *i*, PCR_i_ is the log odds of PCR prevalence, and δ_i_ is the log odds ratio of microscopy to PCR prevalence. δ_i_ is defined as:

$$\delta_{i}=\delta_{i}^{'}+\beta_{0}({\text{PCR}}_{i}-\bar{\text{PCR}})$$

where PCR is the mean survey PCR prevalence, β_0_ is a coefficient shared across all surveys describing how log odds of microscopy prevalence varies with log odds of PCR prevalence, and δ_i_ʹ is a survey-specific intercept coefficient describing the effect of PCR prevalence on microscopy prevalence. This model structure allows δ_i_ to vary between surveys, with β_0_ controlling the extent of this variation. This model was fitted within a Bayesian Markov chain Monte Carlo based framework, implemented in JAGS.^21^ Further information on model fitting is available in appendix 1 (pp 2–3) and the statistical analyses and code implementing the model is available on GitHub.

### Historical and current regional transmission intensity stratification

Surveys done in Africa were geolocated and prevalence estimates (aggregated to the administrative unit 1 level, which represents the highest level of officially delineated area within a specific country) from the Malaria Atlas Project^22^ (MAP) were used to characterise current and historical transmission intensity of the region that each survey belonged to. We distinguished between local malaria transmission (defined by the prevalence recorded in each survey and hereafter referred to as survey PCR prevalence), and malaria transmission at the regional level (reflecting broader patterns of transmission and hereafter referred to as regional prevalence, which represents prevalence averaged at the administrative unit 1 level). This regional-level transmission represents the average of a heterogeneous mixture of higher and lower-transmission areas, and has relevance to local transmission because factors such as human movement patterns and circulating parasite genetic diversity are often similar across nearby settings in the same region, even if transmission levels differ markedly.^23^, ^24^ Regional transmission levels (both historical and current) were then used to stratify each study into one of three transmission archetypes. (1) Historically high and currently high refers to areas with historically (defined as 15 years previous to the date of the survey) high transmission intensity (>15% slide prevalence in children aged 2–10 years) and remain so at the time of the survey; (2) historically high and currently low refers to areas of historically high transmission intensity that have declined in the 15 years previous to the date of the survey to low levels (<15% slide prevalence in children aged 2–10 years); and (3) historically low and currently low refers to areas with historical and current low transmission (<15% slide prevalence in children aged 2–10 years).

Where MAP estimates were unavailable (dates earlier than 2000), it was assumed that the year 2000 was reflective of historical transmission intensity because the substantial increase in international financing for malaria control occurred from 2000 onwards (approximately a twentyfold increase between 2000 and 2015).^25^ Separate Bayesian log-linear regression models were then fitted to data from each transmission archetype to assess the effect of historical and current transmission intensity on the prevalence ratio.

### Estimation of contributions to onwards transmission

We integrated the results of the regression modelling described earlier (which provides an estimate of the proportion of infections that we would expect to be submicroscopic given an estimate of microsopically detectable malaria prevalence) with estimates of infectivity for submicroscopic and microsopically detectable infections to estimate the potential contribution of submicroscopic infections to transmission across different settings. Estimates of comparative infectivity of microscopically-detectable infections versus submicroscopic infections (hereafter referred to as the infectivity ratio) are variable, ranging from a 2× to a 20× difference.^26^, ^27^ We, therefore, explored three scenarios in which microscopically-detectable infections were 2×, 5×, or 20× more infectious to mosquitoes than submicroscopic infections. Proportional contribution to transmission by submicroscopic infections was calculated as:

$$\frac{{\text{LM}}_{-\text{ve}}}{({\text{LM}}_{+ve}\times \text{infectivity ratio})+{\text{LM}}_{-\text{ve}}}$$

where the relative infectiousness of submicroscopic infections (the prevalence of which is denoted by LM_–ve_) is set to 1, and the infectivity ratio (either 2, 5, or 20) is a multiplicative factor reflecting the fact that microscopically detectable infections (prevalence denoted by LM_+ve_) are more infectious. The equation's denominator reflects total onwards transmission occurring within the population, the numerator the amount of transmission attributable to submicroscopic infections. These analyses assume that submicroscopic and microscopically infected populations do not differ in other factors that are likely to influence transmission (eg, age and mosquito exposure).

### Role of the funding source

The funder of the study had no role in study design, data collection, data analysis, data interpretation or writing of the report.

## Results

**Figure 1:**
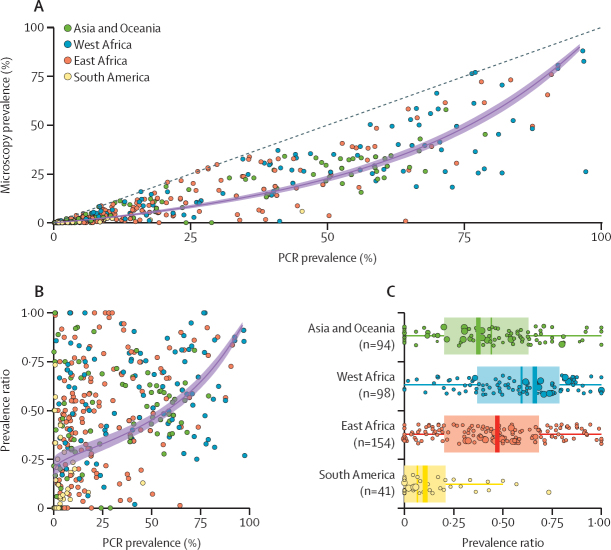
Prevalence of infection by PCR *vs* microscopy in 267 prevalence survey pairs and model fits Bayesian Markov chain Monte Carlo methods were used to fit a linear relationship between PCR prevalence and microscopy prevalence on the log odds scale. (A) 387 microscopy and PCR prevalence surveys were identified in this study and previous systematic reviews. The fitted model relationship (purple line) and the 95% credible interval of the mean (light purple shaded area). (B) The prevalence ratio (ie, the proportion of PCR positive individuals also detectable by microscopy) according to underlying PCR prevalence for each of the 387 survey microscopy–PCR pairs (points) used to fit the full model. The estimated mean prevalence ratio (purple line) and 95% credible interval of the mean (light purple shaded area) are also shown. (C) Box plot of the prevalence ratio disaggregated by global region. For each region, the size of the point reflects the number of individuals tested by microscopy and PCR. Thick coloured bar on the box plot represents the weighted mean prevalence ratio for each global region. Thin line indicates the median, box indicates IQR, and whisker limits span 1·5× the IQR.

4893 potentially eligible studies were identified in the systematic review update and 1768 duplicates were excluded, leaving 3125 studies for screening. After screening titles and abstracts for relevance, 520 studies were kept for full-text evaluation, of which 121 were included. These 121 studies, alongside 45 identified during previous systematic reviews,^14^, ^15^ yielded 551 datapoints comprising distinct cross-sectional surveys in which surveyed individuals had malaria infection assessed by both PCR and microscopy. The number of prevalence survey pairs is greater than the number of included studies because many studies presented results from multiple different locations. 164 of these 551 datapoints were from cross-sectional surveys done in a specific age-group (0–5 years, 6–15 years, and >15 years) and were analysed separately; 387 datapoints were from cross-sectional surveys done in populations that spanned more than one age-group (appendix 1 p 4; appendix 2). Across these data (n=387) included in our primary analyses, microscopy detected 44·9% (95% CI 42·0–47·8) of all PCR-detectable infections, although this varied across settings (figure 1A). In a small number of instances where the number of microscopically detected infections was higher than those identified by PCR (n=10), the prevalence ratio was adjusted to 1, and this adjustment does not qualitatively alter the results described here (data not shown).

We fitted a Bayesian log-linear regression model to both data collated in previous reviews and data newly collected in this study and found no difference in the relationship between PCR and microscopy prevalence (appendix 1 pp 5–6). Some more flexible model structures were also fitted to the data and a log-linear model provided the best overall fit (appendix 1 p 7). The prevalence ratio (defined as the proportion of PCR positive infections also detectable by microscopy) increased as malaria transmission (measured by survey PCR prevalence) increased, indicating a declining proportion of submicroscopically infected individuals. An average of 60–70% of infections were submicroscopic in the areas of lowest PCR prevalence, but only 10–20% were submicroscopic in the highest prevalence areas (figure 1B). There was a small but significant effect of sampling season after controlling for survey PCR prevalence (ANOVA, df=1, p=0·0017; appendix 1 p 8), with submicroscopic infections less common during the wet season than the dry season. There was also a significant effect of PCR methodology (ANOVA, df=4, p=0·038; appendix 1 p 9), with the prevalence ratio marginally lower in surveys using quantitative PCR (qPCR) and RT-PCR to determine infection status. Scanning a higher number of microscopy fields to determine the presence or absence of infection was also significantly associated with the prevalence ratio increasing (ANOVA, df=1, p=0·0053).

**Figure 2:**
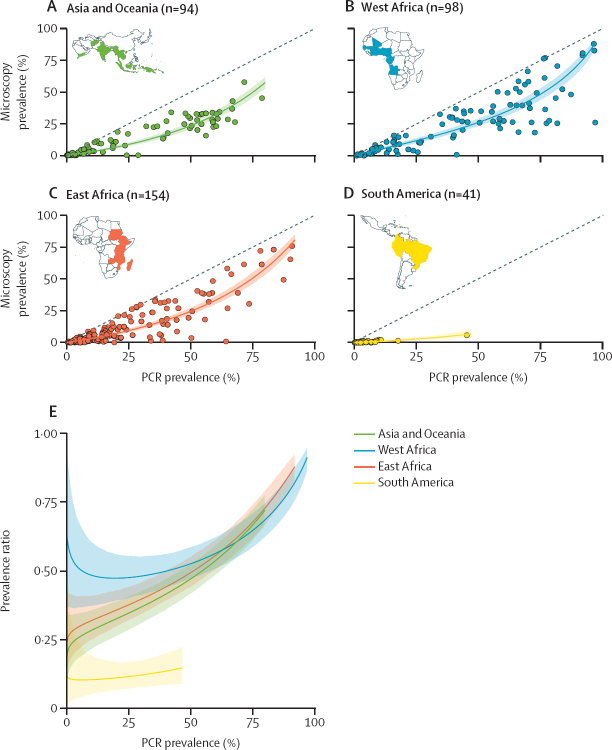
Global variation in the prevalence ratio and the relative size of the submicroscopic reservoir Microscopy and PCR prevalence in included surveys (points), the model-fitted relationship (coloured line) and 95% credible interval (shaded area) for Asia and Oceania (A), west Africa (B), east Africa (C), and South America (D). (E) The model-fitted average microscopy: PCR prevalence ratio by PCR prevalence for each of the four regions (coloured line) and 95% credible interval (shaded area). Coloured countries on each regional map indicates countries for which studies were identified during the systematic review.

Grouping surveys by global region (west Africa, east Africa, South America, and Asia and Oceania) revealed marked geographical variation in the prevalence ratio (ANOVA, p<0·0001, df=3), being lower in South American surveys than all other regions (Tukey's HSD, p<0·0001 for all pairwise comparisons) and higher in west African surveys than all other regions (Tukey's HSD, p<0·0001 for all pairwise comparisons; figure 1C). To examine these differences in more detail, we fitted separate Bayesian log-linear models to the data from each global region and assessed the modelled prevalence ratio across the range of transmission intensities found in each (figure 2A–D). These results revealed that the prevalence ratio in surveys from South America was lower (ie, more infections were submicroscopic) than would be expected based on their respective transmission intensities alone, and consistently lower than all other global regions (figure 2E). Across all settings, nested PCR predominated as the methodology used, although South America had higher levels of qPCR usage than other settings. No significant variation in which microscopy methodology was used between regions was observed (appendix 1 p 10).

The majority of South American surveys had been done in areas marked by historically low transmission. We, therefore, investigated whether a high proportion of submicroscopic infections (low prevalence ratio) might be observed in areas with similarly historically low transmission in Africa. Our results indicated that both regional historical prevalence (in the year 2000) and current prevalence (both averaged over the administrative unit 1 level) were significant predictors of the prevalence ratio when controlling for survey PCR prevalence (ANOVA, p<0·0001 for regional historical prevalence at the administrative unit 1 level, p=0·042 for regional current prevalence at the administrative unit 1 level), suggesting that historical transmission levels, in addition to current transmission levels, are an important determinant of the submicroscopic reservoir size.

**Figure 3:**
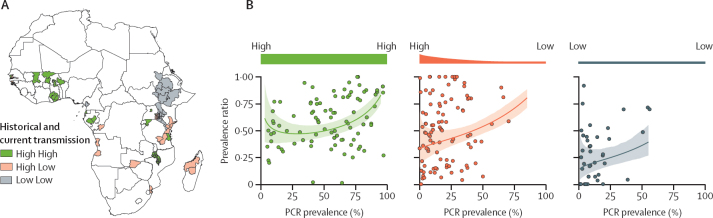
The effect of historical and current transmission intensity on the prevalence of submicroscopic malaria infection in Africa (A) Map detailing the African countries and associated administrative unit 1 level regions for which prevalence surveys were identified, as well as their assigned transmission archetypes based on historical and current transmission intensity (high high=historically high and currently high; high low=historically high and currently low; low low=historically low and currently low). (B) The prevalence ratio of surveys in each transmission archetype (points; n=90 for high high, n=99 for high low, and n=40 for low low), and the modelled average prevalence ratio (coloured line) with 95% credible interval (shaded area).

We next classified each survey in our review from Africa into three transmission archetypes on the basis of the historical and current levels of transmission at the administrative unit 1 level (figure 3A) and fitted Bayesian regression models to each. The results were concordant with those from the ANOVA, with African surveys in regions with both historically and currently low transmission (n=40; Sudan, Ethiopia, and parts of Kenya and Tanzania) having on average a lower prevalence ratio (more submicroscopic infections) than other currently low endemicity areas in Africa where historical transmission was high (n=99) and settings where both historical and current transmission levels were high (n=90; figure 3B). There was no evidence of systematic differences in which PCR and microscopy methodologies were used across different transmission archetypes (appendix 1 p 11) and the observed results were robust to the choice of stratification threshold used to define high and low levels of transmission (appendix 1 p 12).

**Figure 4:**
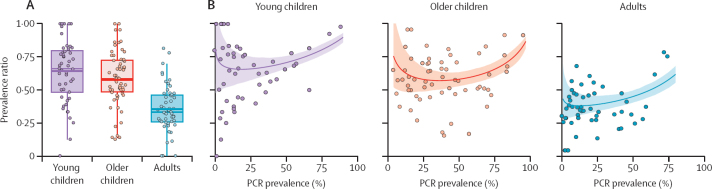
The influence of age on submicroscopic malaria infection (A) Box plot of age disaggregated prevalence survey data for young children (0–5 years, purple points, n=49) older children (6–15 years, pink points, n=62), and adults (>15 years old, blue points, n=53). For each age group, the size of the point reflects the number of individuals tested by microscopy and PCR. Thick coloured bar on the boxplot represents the weighted mean prevalence ratio for each age group. Thin line indicates the median, box indicates IQR, and whisker limits span 1·5× the IQR. (B) The prevalence ratio in surveys where age-disaggregated data (points) were available by age group, showing the fitted model relationship (coloured lines) and the 95% credible interval (shaded areas).

We also collated surveys carried out in specific age groups and defined three age-based categories: young children (0–5 years) giving 49 prevalence survey pairs, older children (6–15 years) giving 62 prevalence survey pairs, and adults (>15 years) giving 53 prevalence survey pairs. The prevalence ratio varied significantly between age groups (ANOVA, p<0·0001, df=2), and was significantly lower in adults (indicating a greater proportion of submicrosopic infections) than in young children (Tukey's HSD, p<0·0001) and older children (Tukey's HSD, p<0·0001; figure 4A). Fitting the Bayesian regression model separately to the data for each age group highlighted that the increased prevalence ratio observed in young children and older children compared with adults was less pronounced in higher-transmission settings. In high endemic areas with 70% overall PCR prevalence, the prevalence ratio for young children was predicted to be 1·42× that of adults, but 1·92× at low endemic areas with 10% overall PCR prevalence (figure 4B). A similar result was observed for adults and older children, suggesting genuine differences in submicroscopic epidemiology both between age groups and across transmission settings.

**Figure 5:**
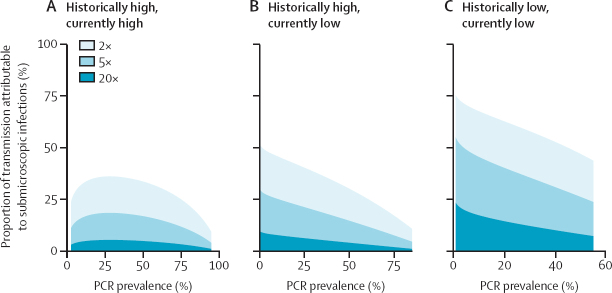
The potential contribution of submicroscopic infections to onwards transmission according to current and historical transmission intensity Potential contribution of the submicroscopic reservoir to onwards transmission for each of the transmission archetypes: historically high and currently high (A), historically high and currently low (B), and historically low and currently low (C) if microscopic infections are either 2×, 5×, or 20× more infectious than submicroscopic infections.

We explored how the contribution of submicroscopic infections to onwards transmission might vary across settings characterised by different historical transmission patterns, using a range of estimates for the comparative infectivity of submicroscopic and microscopically detectable infections. We estimate that in transmission settings characterised by both historical and current low levels of transmission, submicroscopically infected individuals could account for 17·5% to 68·0% of onwards transmission (figure 5C). By contrast, our results suggest the contribution of the submicroscopic reservoir to transmission is less important (although not negligible) in settings where transmission has only recently declined (figure 5B), ranging from 7·8% to 46·0% depending on assumed comparative infectivity.

## Discussion

Considerable debate surrounds the importance of the submicroscopic reservoir to malaria control efforts and whether it needs to be targeted by interventions,^19^ particularly in areas of low transmission. Disaggregating the now larger quantity of available data (551 prevalence survey pairs from 44 countries) has given insight into the complex relationships underlying the global pattern of submicroscopic occurrence. This insight has facilitated a more refined evaluation of when and where submicroscopic infections are likely to be most prevalent and who is most likely to harbour them. Our work suggests that some of the differences observed in the size of the submicroscopic reservoir can be explained by differences in historical patterns of transmission and the age profile of the infected population. Moreover, although previous work has generally noted the potential relevance of submicroscopic infections in low-transmission settings,^28^ our results suggest that this relevance is likely to be highly context dependent, potentially warranting different approaches to the control of submicroscopic infections in different locations.

Both increasing age (independent of exposure) and increased immunity (due to previous exposure, which also increases with age) have been linked to lower parasite densities.^29^ Our results highlight the importance of both these individual-level factors (such as age) as well as setting-specific factors (such as historical patterns of transmission) in determining the size of the submicroscopic reservoir. However, an important caveat to these findings is that there were insufficient data to examine the role of these factors simultaneously. The average age at which an individual is infected is typically higher in low-transmission settings;^30^ therefore, a greater proportion of infected individuals in all-age surveys would be expected to be adults. It is also possible that systematic biases in the age of surveyed populations might exist between geographies or transmission archetypes. Our results surrounding past transmission history might then be confounded by differences in the average age of infection across these different settings. However, the age distribution of malaria infection appears to adapt fairly rapidly to reflect changes in transmission, with infection profiles shifting to older individuals as transmission decline, a feature observed in both clinical cases^31^ and infection in the wider community.^32^ Although this observation suggests that the difference in infected population age profile between surveys from historically low and currently low settings might not be substantial, we are unable to conclusively disentangle the potentially confounding role of age in our analyses of transmission archetypes (and global regions). There is also scope for residual confounding from variation in other factors such as microscopy or PCR methods across locations, although our results collating methodologies used across regions and archetypes suggest this might not be substantial. Although our global regional analyses showed that South American surveys were more likely to have used qPCR (a more sensitive diagnostic), our analysis of transmission archetypes revealed no systematic variation in the PCR methodologies used across archetypes. More broadly, it is also important to note that the data collated here represents cross-sectional surveys of locations that have not necessarily been sampled at random (and instead might be biased towards established research sites), which could introduce systematic bias into the findings.

Another potential limitation is the strong geographical bias in our transmission archetype stratification, which precludes exact determination of the extent to which variation in submicroscopic malaria is driven by geography compared to transmission patterns. The majority of surveys assigned to the historically low and currently low archetype are from east Africa, while the majority of surveys in the historically high and currently high archetype are from west Africa. The observed results across transmission archetypes could, therefore, be reflecting geographical variation rather than variation driven by past transmission history. However, the fact that the proportion of infections that are submicroscopic in historically high and currently low settings (a strata also predominantly composed of studies from east Africa) was consistently lower than that observed for historically low and currently low settings, together with the results observed for South America, provide tentative support for an effect of transmission history on the size of the submicroscopic reservoir, independent of variation due to geographical location.

Several hypotheses could explain these results, including various haemoglobinopathies and human genetic traits that have been linked to lower average parasite densities.^33^ Parasite-related factors could also account for the results observed here, such as systematic variation across locations in asexual blood stage multiplication rate of *P falciparum*^34^ or selective pressures that vary with transmission intensity. Previous work has suggested that high-transmission settings might select for parasites with high replication rates and virulence (to outcompete other co-circulating *P falciparum* strains), whereas low-transmission settings might select for non-virulent parasites with lower rates of replication better able to persist and avoid causing symptomatic infection (which would prevent drug exposure but be more likely to present submicroscopically).^35^ Lower genetic diversity (resulting in more rapidly acquired immunity to local parasite clones) might also contribute to the observed results. It is also not possible to definitively preclude a role for systematic variation in diagnostic quality across settings, although analyses have found that microscopy quality does not vary systematically with transmission intensity.^9^ Although our results highlight that PCR methodology does significantly influence the prevalence ratio, systematic variation in methodological quality across transmission archetype settings was not observed.

Our analyses revealed a significant influence of seasonal effects on submicroscopic carriage, with submicroscopic infections more common in the dry season. This is in keeping with previous work showing that parasite densities rise slightly during the rainy season (even when prevalence does not change significantly).^9^ It is important to note, however, that classification of sampling season was necessarily coarse due to limitations in available data (with information on timing within season being typically sparse). It is possible then that more granular disaggregations might reveal further variation that we have been unable to explore here. Seasonal effects have also previously been shown to play a role in shaping performance of rapid diagnostic tests for malaria^36^ and so future work exploring the factors driving the prevalence of false negatives in these diagnostics (which have shown similar relationships with overall transmission in previous reviews^37^) would also likely be important given their increasing use over microscopy for surveillance and diagnosis of malaria infection.

Our work suggests that the contribution of submicroscopic infections to onwards transmission is likely to be highly variable across settings. However, this analysis is based on the detection of asexual parasites and does not provide direct insight into gametocyte densities. Additionally, due to data constraints, we did not consider a range of relevant factors, such as age profile of the infected population and related skin surface area effects (whereby adults have larger skin surface areas available for biting by mosquitoes compared with children), and adjusting for these would likely increase the contribution to transmission from older children and adults (who are more likely to have submicroscopic infection). The relationship between asexual parasite and gametocyte density is highly non-linear and the distributions of parasite densities in the submicroscopic range can differ substantially between settings.^9^ The proportion of submicroscopic infections might, therefore, not linearly relate to their contribution to onwards transmission. For example, while a membrane feeding study done in Burkina Faso and Kenya (high-transmission settings) found that 45–75% of all mosquito infections were derived from submicroscopic infections,^38^ only 4% of infections arose from submicroscopic individuals in a similar study carried out in Cambodia (a low-transmission setting).^27^ These findings contrast with the predictions presented here and underscore the need to better resolve the relationship between submicroscopic parasite carriage, gametocyte densities, and mosquito infectivity.

Despite their potential relevance to maintenance of malaria transmission, our understanding of submicroscopic infections remains far from complete. Do submicroscopic infections represent a substantial source of transmission and threat to future progress? Do these infections need to be targeted to achieve malaria elimination? Although more work is required, our findings highlight important differences in submicroscopic epidemiology between settings and suggests the absence of a one-size-fits-all solution for malaria control targeting this infection subgroup. Such variation will probably warrant different approaches to malaria control if the infection is to be controlled most effectively in the effort towards elimination.

## Data sharing

Complete details of the code used to analyse the collated data can be found on GitHub.

## Declaration of interests

LCO reports grants from UK Royal Society during the conduct of the study; grants from the Bill & Melinda Gates Foundation and UK Research and Innovation, outside the submitted work. ACG reports grants from the Bill & Melinda Gates Foundation and the Medical Research Council (MRC) Centre, during the conduct of the study; grants from The Wellcome Trust, Medicines for Malaria Venture, Integrated Vector Control Consortium, PATH, National Institute for Health, and personal fees from The Global Fund, outside the submitted work. All other authors declare no competing interests.
